# Accelerating Telemedicine for Cerebral Palsy During the COVID-19 Pandemic and Beyond

**DOI:** 10.3389/fneur.2020.00746

**Published:** 2020-06-26

**Authors:** Hilla Ben-Pazi, Liana Beni-Adani, Ron Lamdan

**Affiliations:** ^1^Multidiciplinary Movement Disorders Clinic, Pediatric Orthopedic Department, Assuta Ashdod, Ashdod, Israel; ^2^Pediatric Neurology, South Region, Leumit Health Services, Ashdod, Israel; ^3^Pediatric Neurology, Ariel, Maccabi Healthcare Services, Tel Aviv-Yafo, Israel; ^4^Faculty of Medicine, Ben Gurion University, Be'er Sheva, Israel

**Keywords:** telemedicine, telehealth, SARS-CoV-2, coronavirus, COVID-19, participatory medicine, digital health, cerebral palsy

## Abstract

The effects of COVID-19 extend beyond the pandemic and are expected to transform healthcare in various ways, many of which remain unknown. With social distancing, telemedicine may become the preferred communication channel between caregivers and patients. Implications for cerebral palsy (CP) children are that this will pose a challenge within this transformation. CP, as a discreet entity, is not considered a risk factor. However, specific comorbidities in individuals with CP, such as chronic lung disease, are known as COVID-19 risk factors. The overall risk for the CP population is probably a factor of age and comorbidities. Staying at home for CP children is both a challenge and an opportunity. Escalation of behavioral conflicts or improved participation and equality within the household may emerge. Interestingly, restricted mobility for the general population narrows existing gaps of ambulation. Telemedicine is the primary way of providing services for chronic conditions during the pandemic and is expected to expand beyond pre-Coronavirus era use. The advantages of telemedicine vary, more so during pandemic times, according to severity, restrictions, and availably of telemedicine. A multidisciplinary therapeutic presence is more accessible with telemedicine, bringing together various specialties and approaches to the child's natural environment. Accessible, continuous care is expected to lower comorbidities, as demonstrated for other chronic conditions. Enhanced monitoring is crucial for younger children as devastating complications, such as hip dysplasia, could be minimized. Last but not least, we will discuss digital health care as an accelerator for participatory medicine, including networked patients and families, as responsible drivers of their health as full partners.

## Introduction

Telemedicine—providing healthcare from a distance—is being exponentially transformed during the Coronavirus pandemic. Telemedicine is traditionally subdivided into being synchronous or asynchronous. Synchronous is defined as interactive video connections transmitting information in both directions simultaneously. Asynchronous telemedicine describes the store-and-forward transmission of medical data in separate time frames (1). Telemedicine services scale up with global emergencies, such as the SARS epidemic in 2003 in China (2). Outside of crisis situations, the overall uptake of telemedicine has been slow and fragmented (3). The advantages of telemedicine became pronounced when it was harnessed to overcome the necessary COVID-19 distancing (4). As the number of COVID-19 cases increases in each country so too does the population's interest in telehealth (5). Acceptance of video-conferencing replacing in-person communication during the pandemic (6) is expected to transform services, especially healthcare.

There are three overlapping periods to consider during the outbreak: early, late, and post-pandemic. Most western countries are currently in the early phase, escalating existing available digital services and attempting to mitigate regulatory barriers (7). This requires abilities to rapidly expand telemedicine capabilities, increase providers' capacities, and update clinical workflows (8, 9). This quick integration is challenging for other, more conventional systems, as it takes 12–15 encounters for a provider starting tele-clinics to feel comfortable using in the ambulatory setting (9). The development of new products and services requires time, even now, with accelerated modes of funding and regulations (10). During the late phase, we expect that novel services and digital devices on the verge of development will enter the market and will be adopted for early use (11). As for the post-pandemic period, the healthcare systems will adopt innovations that proved real value for regular use. While expanding healthcare developments focuses on the current infectious patients, the revolution will also expand to non-infectious care of chronic patients.

This review will focus on telemedicine for children with CP during the outbreak and beyond. We will explore the challenges and opportunities of telemedicine in the CP arena, including practical tips (Table 1). Since clinicians' acceptance plays a crucial role in telemedicine integration (12), we share specific recommendations (Table 2) covering telemedicine set-up, patient examination, and clinical considerations for children with CP, and we do this also beyond standard recommendations for patients with movement disorders (13) and other conditions (9, 14).

**Table 1:** Challenges, opportunities, and tips for CP-focused telemedicine.

	**Challenge**	**Opportunity**	**Tips**
Accessible tele-clinic	Requires video conferencing skills/the child may have difficulty following	Less travel and work loss Functional assessment in the natural environment	Physical distancing is not emotional distancing Emphasize professional look, use reassuring mimicry, manipulate tone to convey the message Confirm that the child and family hear and see you especially during the discussion and recommendation
Continuous therapy	Less physical interaction with	Continuous, accessible, functional	Focus on longitudinal care plan per patient, planning several steps ahead Ask to document outcomes using synchronous and asynchronous (video clips) telemedicine Ensure proper reimbursement
Prevention	Increases care giver responsibility	Less travel for clinical appointments	Proactively initiate tele-follow-up to populations at risk to actively survey their condition and intervene appropriately to prevent deterioration Inform about specific risks with practical recommendations for homebased monitoring Point out red flags for deterioration
Multidisciplinary clinic	Requires more time and effective video- conferencing skills	Multiple specialists Child less intimidated	Use tele-clinics for an inclusive approach. Try to include both parents, community/school therapists, and a multidisciplinary medical team In the essence of time, focus each visit on one functional problem. Send background materials before the visit The deliverable of the multidisciplinary clinic should be a personalized, tailored, coordinated plan, including medical recommendations, physical treatments (PT, OT, ST), and educational-psychological therapies
Participatory medicine	Requires caregivers' responsibilities	Patient centric treatment	Focus on the child's and family's function goals and quality of life Plan a series of discrete steps to reach the discrete endpoint Provide indicators of success and hurdles

**Table 2:** Tele medicine for CP-Practical recommendations.

	**Unique issues**	**CP emphasis**
Set-up	Visual impairment	Face should fill at least 1/3 of screen width White background Enhance mimicry
	Auditory impairment	Check that the sound is loud and clear several times during the conference Enhance vocal cues and variability
	Family and child	Wide shooting angle to include all participants or using different screens for each participant
	Comfortable environment	As the online visits may be long to ensure that all are comfortably seated
Examination	Physical disability	Two adults (parents) necessary for video examination one for manipulation and other for video recording
	Relaxing	Postponed stressful topics after the exam to ensure maximal relaxation Painful items should be postponed to the end of the exam
	Range of motion	Make sure that camera is 90 degrees to the angle assessed Consider requesting parents to take pictures and sending (asynchronous for formal ROM assessment
	Intellectual disability	Consider exposure of intimate undressing as poses an educational conflict
Clinician considerations	Emotional stress	Assess and treat the emotional aspects
	Chronic deteriorating condition	Follow up
	Recognizing problems	State demonstrate clear red flags
	Communication availably boundaries	Set clear communication rules on how to contact during routine and emergency

## Accessible Care

Telemedicine has already been demonstrated as being useful for chronic conditions (15), including movement disorders (16), but it until now has been underutilized for standard care despite the ubiquitous smartphone platform (2, 13). Technical hurdles on the patient's end could be addressed by virtual front desk conducting a pre-test. The gap seems more remarkable considering the limited and unequal accessibility for patients with CP compared to general population, including their need for mediators to access most tele and non-tele services. Paradoxically, tablets and smartphones improved accessibility to patients with CP to a multitude of online or application mediated activities, schooling, and entertainment (17–20).

Hospital telemedicine services are currently focused on reducing infection spread by providing tele-emergency services (21). Primary tele-healthcare services available before the pandemic (19) are expanding to manage the majority of home-isolated Coronavirus patients addressing diagnostic and treatment aspects (3, 8, 22–24), and some institutions show a significant increase in tele-consultations compared to pre-pandemic era (9). Left with a choice of not-consulting at all vs. using telemedicine platforms, the “Philadelphia group” has clearly shown that patients preferred to use tele-visits rather than avoid clinical consultations (9). Individuals with CP are not specifically at risk from COVID-19, unless they suffer from comorbidities such as pulmonary disease or elevated blood pressure (25). However, to minimize exposure, many children stay home away from the daily treatments and services provided in rehabilitation schools and clinics. Elective consultations and paramedical treatment, including utilization of heavy equipment, walking aids, hydrotherapy services, and more, are diminished if not ceased. While much emphasis is on medical or physical risk and deterioration, the emotional comorbidity is relatively neglected. Individuals with chronic disabilities, including CP, have higher rates of anxiety and depression (26), and this is expected to worsen during the pandemic, even more so when most of them are far from their professional therapists. Increased stress worsens hypertonia and impairs daily life. Lack of physical treatment undermines function even further.

The result for the last months has been in most countries, that complex medical care for children with CP, that traditionally take place in outpatient clinics and institutions cannot be accessed. Telemedicine is thus one of the preferred and most accessible platforms for these children. Many children use tablets and computers almost independently or with minimal aid for social communication and learning; some can use smartphones. Proper instruction on how to use familiar technology for CP- tele-healthcare can therefore minimize gaps in physical and mental health when matching clinical needs to available opportunities (Tables 1, 2).

## Continuity of Care

Children with CP require continuous care, both paramedical and medical (27). Many patients take chronic medications, some of which need more monitoring than others, including blood tests and clinical examinations. Some patients are treated with Intrathecal Baclofen pumps and need routine refills of the pump to avoid withdrawal symptoms and increased spasticity. Following selective dorsal rhizotomy (SDR), continuous massive physiotherapy is needed to decrease tone and increase functionality. Serial Botulinum toxin injections are preplanned as a program of long-term treatment to improve the functional range of movement (28).

During the acute phase of the pandemic, many hospitals closed outpatient clinics and canceled so-called elective appointments, and remote platforms of urgency grading were initiated (9, 23, 29). While necessary procedures mandating face-to-face interaction should not be canceled, consultation before and after the procedure may be performed by telemedicine (7, 9). Tele-clinics can replace many of clinical encounters, such as adjusting medication, orthopedic monitoring, and neurosurgical evaluation (13, 17). During routine follow-ups, we check by observation motor symptoms, stiffness, and spasticity as compared to “regular status,” range of motion of limbs (any change in range, position), and increased difficulty in dressing/undressing. Non-motor issues such as pain, sleep, feeding, and communication could be easily evaluated by telemedicine in individuals with CP (16, 17, 20). Finally, it is required to address the relevancy and urgency of medical treatment in any chronic patient. Spasticity related emergencies are not common, and most surgical procedures in patients with CP are considered elective. Telemedicine can assist in identifying and prioritizing medical assistance in surgical (9) emergencies (neurosurgery includeed) (29). For example, neurosurgical services prioritized according to urgency in three different classes (immediate treatment, treatment within 7–10 days, and treatment within a month) (29). Tele-Guide-lined and Structured Continuous Care (TGSCC) can be provided for each patient, shared among healthcare providers, with practically no limitations of geographical distances (9). During the acute phase of the pandemic the main contribution of TGSCC is to maximize family aid and interference guided by professionals.

During post-Corona era, this tool could still maximize monitoring of the chronic patient and provide consultation and follow-up as for adjusting physical therapy, medication management, and psychosocial management whenever traveling and face-to-face treatment is not mandatory (14). Novel post-Corona services may enable prioritizing necessary procedural actual visits, using accessible telemedicine to focus on the specific inquiry for changes over time from baseline status.

## Prevention

Non-progressive neurological damage defines CP but does not imply a uniform, unchanging clinical behavior. Individuals with CP exhibit alterations in muscle tone with resultant changes in tendon-muscle unit length over time (27, 28). Muscle tone increases with stress, interfering with activities-of-daily-living, including dressing, feeding, hygiene care, and speech and may also limit the range of motion (30). Ultimately, increased tone leads to skeletal changes, such as hip displacement and spinal scoliosis, that may eventually cause pain and dysfunction, impacting health, wellness, and quality-of-life. While immediate consequences of tone changes may be monitored and treated, the long-term effects are very gradual, subtle, and challenging to recognize. Routine monitoring was found as the most effective way to eliminate contractures and hip displacement (31), with home visits being more effective than scheduled outpatient follow-up (32–35).

In many countries, most healthcare resources during the pandemic are diverted to COVID-19-related needs and less to routine care and prevention. If this trend continues, care of the CP population may be compromised. With less therapy, there is a substantial risk of irreversible contractures and the development of deformities that may eventually require surgical intervention. Failure to follow hip surveillance guidelines may further jeopardize the care. It is difficult to estimate the length of time after which the reduction in treatments and clinical and radiographic follow-up will begin to instigate deterioration. Telemedicine is an effective and accessible platform to enable attention, follow-up, and treatment of children with CP. Physicians and therapists should use telemedicine and encourage families to tele-communicate with medical and paramedical staff led by both pre-planned and on-demand principles to prevent deterioration and maintain follow-up.

CP-UP provided ample evidence that surveillance based on the CP type, age, Gross Motor Function Scale (GMFCS) score, and findings on physical exam minimize the risk of hip displacement (1, 36). Tele-monitoring applications can transform these protocols into a decision-support-system combining medical history, physical examinations, and radiographs to provide follow-up recommendations for early detection and prompt interventions (37, 38). Coronavirus pandemic increased the use of home-based tele-monitoring for COVID-19 patients (3–5, 8, 10). We expect these services to expand to broader use beyond the pandemic and harness them to bridge the gaps of essential routine monitoring for populations at risk, for other functional assessments in the natural environment such as gait monitoring as a screening tool for referrals to gait laboratories and remote rehabilitation monitoring. These services are yet to be developed and would need to determine the best user experience and guidance to perform orthopedic goniometry and radiological surveillance. Tele-hip monitoring has specific challenges such as camera position in a 90-degree angle to reflect the true hip abduction and appropriate protection of sensitive exposed areas. Improved orthopedic (hip surveillance) and functional (gait recording) monitoring may lead to minimization and even prevention of deterioration in children with CP.

## Multidisciplinary Approach

The healthcare needs of the CP population are complex (27, 28). A multidisciplinary approach has therefore gained popularity in recent years, with well-documented benefits that include improved coordination of care and time use optimization for patients and their caregivers (17, 30). However, these multidisciplinary setups may become impossible at times when physical distancing is required to limit exposure during the pandemic, as contact between patients and caregivers is greater than regular clinics. To maintain the high quality of multidisciplinary clinics and their achievements over time for patients with CP, telemedicine may become an even more promising platform. Telehealth may especially be relevant as challenges for these patients and families are intensified by school closures, crowding at home, and decreased access to healthcare and therapies. Telemedicine used as a multidisciplinary tele-meeting consumes less time and effort for the families and is more effective for Tele-Guide-lined and Structured Continuous Care (TGSCC). It maintains the added value of multiple specialists assembled without jeopardizing the possibility of discussion and re-evaluation based on real time interaction with the patient and family with all healthcare providers, patients, and caregivers being in their protected environment (18, 39, 40). Such tele-contact also obviates the need for the use of personal protective equipment such as gloves and face masks, limits the risk of viral spread, and offers patients a more natural environment without compromising health care personnel and patients' safety (21).

In the post-pandemic period, we expect telemedicine to become more coordinated and patient-centric, providing tools of assessment and reimbursement of multidisciplinary tele-care (9, 12, 23, 24). This challenge extends beyond the technical platform of bringing together multiple people for a video conference (41).

## Participatory Medicine

Medical conduct is moving from paternalistic conduct to participatory medicine (42). The conservative way of managing the patient is transforming as technology-enabled families are connected via social media and are better informed than in the past by available self-research. The internet and tele-communication era lead to shifting to a more mutually inclusive healthcare (43, 44). Predictive, Personalized, Preventive, and Participatory (4P) medicine is advantageous with telemedicine tools (44). This movement, which used to exist in the periphery of medical care, is currently expanding, public healthcare awareness is rising, and participatory medicine is becoming accepted as citizens are requested to report their health status. Corona pandemic introduces innovative and crowdsourcing technology utilizing participatory medicine. People choose to share their health status to help others on a broader scale via social media, even more than was available in the pre-coronavirus pandemic (9, 10). Some countries, like Taiwan, used big data from citizens to track and control COVID-19 spreading (24). This trend poses privacy challenges but introduces therapeutic opportunities that will extend beyond the current crisis (7, 9).

Participatory medicine is especially important in chronic disorders (4, 41). In terms of research, a patient-centric approach is on the rise when it comes to children with CP (13, 45, 46). The comparative effectiveness of interventions, physical activity, and understanding aging were leading themes, highlighting the need to focus on longitudinal research that includes outcomes related to participation and quality-of-life (46). We believe that implementation will grow with telemedicine expansion. This is an opportunity for a therapeutic presence shift in CP: from the hospital to home, from somatic emphasis to a holistic approach, and from the physical examination to functional observation in the natural environment. Moreover, telemedicine assists other stakeholders to be involved in the child's care as needed (47).

## Discussion

In this mini review, we covered focused aspects of telemedicine for children with CP and its recent developments during the Coronavirus pandemic. The social, psychological, economic, and health burden of the COVID-19 pandemic on patients with CP and their families is enormous. We believe that teleconsultations can address aspects of continuous CP care, such as managing medications and providing exercises in home environments, with increased participation of patients and families (44–46). Reports have shown that patients prefer visual tele-consults rather than phone or e-mail consultations (5, 7, 9). In some countries, the pandemic has imposed reconsidering telemedicine (21–24), as “locked-down” reality and restricted services expanded for non-urgent and non-infectious treatment (7, 9). In other countries, with different levels of severity and variability in restrictions in face-to-face medicine telemedicine use was more variable. Telemedicine expansion also depended on available technology.

Patients with CP may be clinically dynamic, even though CP is a chronic situation deriving from non-progressive neurological damage. When withdrawn from routine services, physical and emotional difficulties occur, increasing spasticity and causing consequent functional emotional and behavioral deterioration (26, 28, 30, 31). Currently, in our service, to maintain continuity, parents are sharing with healthcare professionals video clips of patients with CP in their home environment standing, sitting, walking, playing, and communicating, each according to her or his functional status.

### Possible Disadvantages and Difficulties of Telemedicine for Patients With CP

Aside from the natural tendency to avoid changes, disadvantages often quoted were concerns regarding less personal approach, patients' security, confidentiality concerns, liability issues, need for medical report integration, decreased reimbursement and questions regarding intellectual property rights (2, 14, 22, 23, 34). This is particularly important with respect to safeguarding children and vulnerable adults. Important possible limitations of telemedicine discussed were verification of patient identity and location as well as a difficulty to obtain informed consent and a secure HIPAA-compliant platform (Health Insurance Portability and Accountability Act) (7, 9). These difficulties, may be overcome with implementing adopted strategies creating adjusted reimbursement methods and re-defining workflows (9, 12). Fortunately, there is a growing modification of the payment policy in response to COVID-19 globally (9, 23). We hope others will follow suit, and equivalent reimbursement would become the norm. Also, “myths” need to be overcome: “patients prioritize relationships over transactional care,” “the physical examination is missing,” “virtual visits are not sufficient,” and more. Currently, not all medical and paramedical staff are familiar with the use of teleconferencing technologies in instructing, monitoring, and diagnosing individuals with CP and their needs. By minimal training this may be overcome (7–9, 13), together with engaging families to use familiar technology, routinely used for social communications, for medical purposes. Many low-income populations do not have computers, smartphones, or software to participate in video conferences; thus, with the expansion of services, providers should consider installing local telemedicine kiosks.

Last but not least, Telemedicine cannot completely substitute face-to-face clinics. However, it is most beneficial where conventional healthcare is lacking or impossible providing accessible, continuous care, with real-time visualization.

### Changing the Concept and Using “Familiar Social Technology” in “Clinical Setup”

Despite telemedicine prospects and the extensive use of technology for social communication, many clinicians and healthcare systems were reluctant to adopt it as a standard way of providing care. This paper has discussed the advantages of telemedicine emphasizing accessibility, its potential to provide multidisciplinary care, and enhanced patient participation. We have also discussed the advantage of real-time visualization of patients and families, regardless of the geographical distance, and the availability of several experts when video conferencing in the same session without travel discomfort or risk. In order to successfully incorporate telemedicine in the post-Corona era, now is the opportunity to open new platforms and workflows of care for the post-Corona era and to address the reimbursement hurdle (e.g., only 20% of states in the US require payment parity between telemedicine and in-person services) (7). The next steps include ensuring equitable access to affordable telemedicine for CP patients and evaluation of quality assurance.

In this mini review, we speculated how telemedicine program growth would develop efficient workflows for individuals with CP, suggesting the implementation of the “Guide-lined and Structured Continuous Care” (TGSCC) platform. Such an approach should be patient-centric, taking into account the ability of the family to increase participation and take more daily responsibility and action, a parameter that has been shown to contribute to non-CP patients effectively (7). An integrated decision–support system should be able to create a workflow allowing informative and visual input from the patient/family, assessing the status and diagnosing possible changes/deterioration, including instructional therapies by tele-guidance, reassuring, correcting and re-evaluating, and allowing access to medical consultation and therapists' guidance as needed. Utilization of services on the tele-media platform for medical consultations for CP may assist in minimizing deterioration and irreversible physical, functional, emotional, and behavioral damage as well as providing reassurance to families.

Further research is necessary to evaluate both medical and psychological aspects of a shift to telemedicine on patients, including CP, as well as on healthcare workers. Controlled studies are needed to evaluate in the long term the impact of clinical practice from an epidemiological point of view. Combining a multidisciplinary approach to the COVID-19 imposed telemedicine should be explored and popularized if proven effective. Improved communication technologies, with good quality of visual and sound data transfer, and re-definition of reimbursement criteria are mandatory components to facilitate high-quality telemedicine for individuals with CP in the future.

## Author Contributions

HB-P: conceptualized, formatted, and drafted the paper. RL and LB-A: contributed to the writing and revised the mini review. All authors contributed to the article and approved the submitted version.

## Conflict of Interest

HB-P is the founder and owner of NeuroCan LTD, a telemedicine company. The remaining authors declare that the research was conducted in the absence of any commercial or financial relationships that could be construed as a potential conflict of interest.
